# Transferrins Reduce Replication of *Chlamydia suis* in McCoy Cells

**DOI:** 10.3390/pathogens10070858

**Published:** 2021-07-07

**Authors:** Leentje De Puysseleyr, Kristien De Puysseleyr, Joanna Rybarczyk, Paulien Vander Donck, Winnok H. De Vos, Daisy Vanrompay

**Affiliations:** 1 Laboratory for Immunology and Animal Biotechnology, Faculty of Bioscience Engineering, Ghent University, 9000 Ghent, Belgium; leentje.depuysseleyr@gmail.com (L.D.P.); kristien.depuysseleyr@gmail.com (K.D.P.); j.rybarczyk@abcheckantibodies.com (J.R.); Paulien.VanderDonck@UGent.be (P.V.D.); 2 Laboratory of Cell Biology and Histology, Faculty of Pharmaceutical, Biomedical and Veterinary Sciences, University of Antwerp, 2610 Wilrijk, Belgium; winnok.devos@uantwerpen.be

**Keywords:** *Chlamydia suis*, swine, transferrin, ovotransferrin, lactoferrin

## Abstract

*Chlamydia suis* (*C. suis*) resides in the intestines of pigs and tetracycline-resistant strains are emerging worldwide. Intestinal infections are often subclinical. However, the gut is regarded as a *C. suis* reservoir and clinical infections have been associated with enteritis, conjunctivitis, pneumonia and reproductive failure. *C. suis* was found in boar semen and venereal transmission occurred. We studied the anti-*Chlamydia suis* activity of ovotransferrin (ovoTF) and bovine lactoferrin (bLF). Pre-incubation of *C. suis* with bLF or ovoTF had no significant effect on overall chlamydia replication (mean fluorescence area) in McCoy cells. The addition of ovoTF to the culture medium had no effect on bacterial replication, but the addition of 0.5 or 5 mg/mL of bLF significantly reduced the inclusion size by 17% and 15% respectively. Egg components are used for cryopreservation of boar semen. When inoculating an ovoTF-containing and *Chlamydia suis-*spiked semen sample in McCoy cells, a significant reduction in inclusion number (by 7%) and overall replication (by 11%) was observed. Thus, we showed that transferrins possess anti-chlamydial activity. Moreover, ovoTF addition to semen extenders might reduce *C. suis* venereal transmission. Further research is needed to unravel the mechanisms behind the observations and to enhance the effect of transferrins on *C. suis*.

## 1. Introduction

The genus *Chlamydia* consists of obligate intracellular bacterial species that cause disease in a broad range of host animals. *Chlamydia suis* (*C. suis*) is considered endemic in the intestinal flora of pigs [1]. Although these intestinal *C. suis* infections are widespread, they usually remain subclinical [2,3]. However, *C. suis* infections in pigs can also lead to enteritis, conjunctivitis, pneumonia and reproductive failure in sows and boars, leading to economic loss [4,5,6]. Moreover, the presence of *C. suis* in pig semen has already been demonstrated and suggested as a possible route of transmission among pigs [7,8,9]. Tetracycline is the main antibiotic used in the treatment of chlamydial infections. However, since 1998, tetracycline-resistant *C. suis* strains have been emerging worldwide [8,10,11,12,13,14,15,16,17]. Alternatives to antibiotics are urgently needed, as antimicrobial resistance is an increasingly important concern for both human and animal health [18]. Furthermore, there is considerable pressure to diminish antibiotic use in animal production. A critical component of the antimicrobial resistance solution is the development of truly novel and innovative alternatives to antibiotics to cover not only the diminishing effectiveness of existing antibiotics but also to support sustainable antibiotic use, preserving them exclusively for treatment of life-threatening infections.

Transferrins might present an alternative to antibiotics. Lactoferrin (LF), ovotransferrin (ovoTF), and serum transferrin, are the most important members of the transferrin family of iron-binding glycoproteins. The function of this protein family is not solely limited to iron-transport [19,20,21].

Lactoferrin is secreted by the mucosal epithelial cells of multiple species, including cows, goats, horses and humans. Consequently, this transferrin is present in tears, saliva, vaginal secretions, semen and, most abundantly, in milk. Lactoferrin is present in neutrophil granules from where it is released upon inflammation. As such, LF is involved in the modulation of the inflammatory response to pathogens [22]. Lactoferrin can bind and sequester lipopolysaccharide (LPS), thus preventing pro-inflammatory pathways, sepsis and tissue damage. However, LF-bound LPS may retain the capacity to induce cell activation via Toll-like receptor 4-dependent and -independent mechanisms [23]. Lactoferrin acts as a primary defense against pathogenic bacteria, viruses, fungi and protozoa. Its function as an antimicrobial protein or peptide is most studied and is based on multiple mechanisms [24]. Lactoferrin has a bacteriostatic effect through iron-binding, leading to iron deprivation of the microorganisms. In addition, LF affects bacteria directly by destabilizing the outer membrane of Gram-negative bacteria through binding of bacterial LPS [25,26,27,28]. Moreover, LF can cause selective permeation of ions through the inner membrane [29], and it interferes with bacterial adherence on host cells through disruption of the bacterial Type III secretion system (TTSS) [24,30,31]. Lactoferrin not only binds endotoxins (LPS), it also influences the release and bioactivity of Shiga toxins from enterohemorrhagic *Escherichia coli* O157:H7 (EHEC) strains [32], and it degrades F4 fimbriae, F18 fimbriae and flagellin of porcine EHEC strains. Lactoferrin can also decrease the number of adherent bacteria to porcine small intestinal epithelial cells [33].

Ovotransferrin is produced by birds and is predominantly present in serum and egg white. Similar to LF, ovoTF has a role in iron transportation and delivery [34]. Moreover, it also exerts antimicrobial effects on Gram-negative bacteria [35] through permeation of the outer membrane followed by selective permeation of ions through the inner membrane leading to disruption of the electric potential [29,36,37]. This bactericidal activity can be attributed to the amino-terminal N-lobe of ovoTF from which the cationic antimicrobial peptide OTAP-92, consisting of 92 amino acids, was derived. Ovotransferrin degrades F18 fimbriae and flagellin, but not F4 fimbriae of porcine EHEC strains, and decreases the number of adherent bacteria to porcine small intestinal epithelial cells [33].

Lactoferrin and ovoTF have already proven their potential to reduce chlamydial infections in vitro and in vivo. Beeckman et al. [38] was the first to demonstrate the inhibitory effect of human lactoferrin (hLF), bovine lactoferrin (bLF) and ovoTF on the infectivity, adhesion and invasion of *C. psittaci* in HD11 chicken macrophages. The same group also successfully used ovoTF spraying to prevent *C. psittaci* respiratory disease in experimentally infected specific pathogen-free turkeys and on a commercial turkey broiler farm [39,40,41]. Farm use of ovoTF: (i) reduced the amount of *C. psittaci* in the air, as demonstrated by bioaerosol monitoring; (ii) prevented respiratory disease during the first half of the brood period; (iii) was associated with a 46% reduction in mortality; and (iv) reduced the antibiotic cost [40].

Currently, the antibacterial activity of transferrins towards *C. suis* has not been investigated. In order to evaluate the use of bLF and ovoTF against *C. suis* infections in pigs, we examined the effect of these transferrins on extracellular *C. suis* and on intracellular replication of *C. suis* S45 in vitro. Additionally, the use of ovoTF to reduce the in vitro infectivity of *C. suis* in spiked boar semen was evaluated.

## 2. Results

To determine the maximal non-cytotoxic concentration of ovoTF and bLF for McCoy cells, a 3-[4,5-dimethylthiazole-2-yl]-2,5-diphenyltetrazolium bromide (MTT) assay was performed. Up to 2.5 mg/mL of ovoTF and 10 mg/mL of bLF did not affect the cellular metabolism at the time points examined. At 24 h and 48 h, 5 mg/mL of ovoTF decreased the cellular metabolism by 50%. At 24 h and 48 h, 10 mg/mL of ovoTF reduced cellular metabolism by 85% and 90%, respectively. Only non-cytotoxic concentrations of 0.5 mg/mL of ovoTF, 0.5 mg/mL of bLF and 5 mg/mL of bLF were further used.

**Figure 1:**
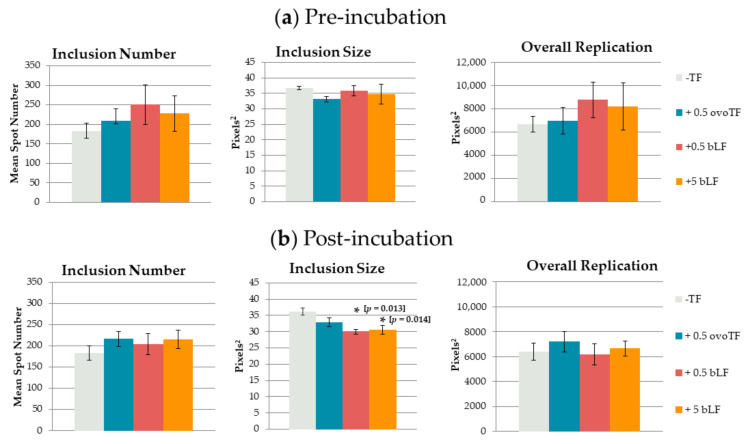
Effect of transferrins (TF) on *Chlamydia (C.) suis.* (mean ± SE). *C. suis* was incubated with ovotransferrin (ovoTF) and bovine lactoferrin (bLF) (**a**) prior to inoculation (pre-incubation) and (**b**) from inoculation onward (post-incubation). Replication of *C. suis* was compared based on inclusion number (mean spot number), inclusion size (mean spot area) and overall replication (mean fluorescent area) in McCoy cells. Significant differences as compared to the control are designated with an asterisk, accompanied by their *p*-value.

To evaluate the effect on extracellular bacteria, *C. suis* S45 bacteria were incubated with transferrins prior to inoculation in McCoy cells. Pre-incubation of *C. suis* with ovoTF or bLF had no significant effect on the inclusion number, inclusion size, or overall replication in McCoy cells (Figure 1). A repetition in time gave the same result: no significant effects were observed (data not shown).

To assess the influence of transferrins on intracellular replicating bacteria, incubation with transferrins was performed post-inoculation (p.i.). The addition of 0.5 or 5 mg/mL of bLF to the culture medium lowered the number of inclusions, as well as the overall replication, albeit not significantly as compared to the control. The inclusion size was significantly reduced by adding bLF to the culture medium. As compared to the control, the inclusion size was reduced by 17% (*p* = 0.013) and 15% (*p* = 0.014) when incubating with 0.5 or 5 mg/mL of bLF, respectively (Figure 1). However, there was no significant dose effect. Supplementing ovoTF to the culture medium had no significant effect on the inclusion number, inclusion size or overall replication. A repetition in time gave the same result, namely only significant effects on the inclusion size when using 0.5 and 5 mg/mL of bLF (data not shown).

**Figure 2:**
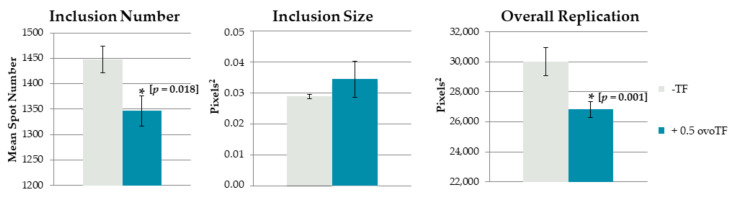
Effect of ovotransferrin (ovoTF) on *Chlamydia (C.) suis*-spiked pig semen. (mean ± SE) *C. suis* was added to negative pig semen and incubated with or without 0.5 mg/mL of ovoTF prior to inoculation. Replication of *C. suis* was compared based on inclusion number (mean spot number), inclusion size (mean spot area) and overall replication (mean fluorescent area) in McCoy cells. Significant differences are designated with an asterisk, accompanied by their *p*-value.

To prevent venereal spread of *C. suis*, an experiment was set up to examine the effect of adding ovotransferrin to *C. suis* spiked pig semen, as chicken egg components are already used for cryopreservation of boar semen. First, all three boars tested negative in *C. suis* real-time PCR and *C. suis* recombinant MOMP-based antibody ELISA. When inoculating the ovoTF treated, or non-treated, *C. suis* spiked semen samples in McCoy cells, a significant reduction in the inclusion number [reduced by 7% (*p* = 0.018)] and overall replication [reduced by 11% (*p* < 0.001)] was observed for the treated sample (Figure 2). A repetition, using semen of another *C. suis* negative boar, gave the same result, namely a significant reduction in the inclusion number and overall replication (data not shown).

## 3. Discussion

The anti-bacterial effect of transferrins has already been described for Gram-positive and Gram-negative bacteria [34,42]. Currently, the anti-chlamydial effect of transferrins has only been demonstrated for the avian pathogen *C. psittaci* [38,39,40] and, more recently, for the human pathogen *C. trachomatis* [43]. Emerging tetracycline resistance in *C. suis* strains in the domestic pig population worldwide [8,10,11,12,13,14,15,16,17] requires the development of alternative preventive strategies or therapeutics. Therefore, we examined the effect of transferrins towards the *C. suis* reference strain S45.

Bacteria were incubated with transferrins prior to inoculation in McCoy cells or from inoculation onward. Pre-incubation of *C. suis* with ovoTF or bLF prior to inoculation in McCoy cells had no significant effect on the number of inclusions, the inclusion size or the overall replication, suggesting no effect on extracellular bacteria. Beeckman et al. [38] performed a similar, but not identical, trial for the avian pathogen *C. psittaci*: pre-incubating 10^8^ Tissue Culture Infective Dose 50 (TCID_50_) bacteria with ovoTF, bLF and hLF followed, by inoculation in BGM cells. They noticed a significant reduction in the infection outcome when pre-incubating *C. psittaci* with 0.5 to 5.0 mg/mL of ovoTF, followed by inoculation in BGM cells. Bovine and human LF had no significant effect on the infectivity of *C. psittaci*. Therefore, in their study, the avian transferrin acted better on the avian extracellular chlamydiae. This might suggest that porcine LF would be better for reducing the infectivity of extracellular *C. suis*. However, this can only be proven by using the identical experimental conditions used by Beeckman et al. [38]. Therefore, a batch of recombinant porcine LF is currently being produced, as described by Dierick et al. [33], to examine this hypothesis in depth by using the previously described method [38]. We already used this recombinant porcine LF (pLF) successfully in in vitro studies with porcine enterotoxigenic *Escherichia coli* [33]. However, recombinant pLF is much more expensive compared to ovoTF or bLF, and thus less attractive for use in the pig industry. One could use native pLF purified from sow colostrum or milk. However, native pLF is commercially unavailable for two reasons: (1) pLF concentrations in sow colostrum and milk are rather low and (2) the ‘successful’ pig breeding programs [44,45] resulted in larger litters but, unfortunately, also in a shortage of colostrum and milk for the piglets, as colostrum and milk production by the sow remained unchanged. This is in contrast with the large availability of ovoTF or bLF from chicken egg whites or cow milk.

Incubation of *C. suis*-inoculated McCoy cells with ovoTF had no significant effect on inclusion number, inclusion size or overall replication. Bovine LF, on the other hand, significantly reduced inclusion size. The number of inclusions and the overall replication of *C. suis* were also reduced by bLF, albeit not significantly. However, no dose effects were observed for bLF. The latter might perhaps be explained by the transferrin receptors present on McCoy cells. In this experiment, effects on intracellular chlamydiae can only occur when ovoTF or bLF are able to enter McCoy cells (mouse fibroblasts), as recently shown by us for bLF in bovine primary rectal cells [46]. The presence or absence of transferrin receptors (intelectin, nucleolin) on McCoy cells might be verified by RT-PCR and immunofluorescence assays but given, bLF has an effect, its receptor must be present. Furthermore, the ovoTF receptor must be expressed on McCoy cells, as our semen experiment demonstrated an effect for ovoTF.

*Chlamydia suis* can be venereally transmitted via contaminated boar semen [7,8,9]. Schautteet et al. [8] examined a case of severe reproductive failure in a Israeli pig farrowing to slaughter farm. The Israeli farm most likely became accidently infected with *C. suis* through the import of boar semen from a German boar insemination center, as *C. suis* was retrospectively isolated from the semen of four out of five boar studs used. National and international trade of boar semen for insemination is common. Semen for artificial insemination is typically obtained from high health and specific pathogen-free herds. They are frequently tested for porcine reproductive and respiratory viruses (PRRSV) and, on occasion, other porcine viruses via PCR to avoid transmission. Screening does not regularly include bacteria and, as far as we know, does not include *C. suis*, which is a relatively ‘new’ emerging pathogen in the pig industry. Furthermore, the addition of antibiotics in commercially available extenders is generally believed to limit or preclude the transmission of infectious bacteria. However, Hamonic et al. [9] have already reported extended boar semen used for artificial insemination as a potential transmission mechanism for *C. suis*. *Chlamydia suis* remained viable and infectious during chilled storage and was globally unaffected by antibiotics (penicillin, streptomycin, gentamycin) in the extender.

Therefore, we evaluated the effects of ovotransferrin on the replication of *C. suis* from spiked pig semen samples in vitro. Ovotransferrin is the second most abundant (12%) protein present in egg whites, following ovalbumin (54%), and is purified from chicken eggs on industrial scale. OvoTF (± EUR 200/kg) is cheaper than bLF (± EUR 800/kg) and thus more attractive to the pig industry. Furthermore, egg material, namely egg yolk, is widely used for boar semen cryopreservation [47]. Incubation of *C. suis*-spiked negative pig semen with 0.5 mg/mL of ovoTF resulted in a significant reduction in the number of inclusions formed during the cultivation of *C. suis* from pig semen in cell monolayers. Moreover, the overall replication of *C. suis* was significantly reduced. These findings are contradictory to the results of the post-incubation experiment with ovoTF, which showed no effect on S45. However, the constitution of the semen sample, for instance the bicarbonate (mainly originating from the seminal vesicles) or salt (epididymal fluid) concentrations, could have influenced the activity of ovoTF in semen. Indeed, Ko et al. [48] demonstrated that the addition of bicarbonate enhanced ovoTF antimicrobial activities against *Escherichia coli* O157:H7. Moreover, bicarbonate, involved in the initial binding of iron to ovoTF, enhanced the activity of ovoTF towards *Staphylococci* species [49]. Additionally, the antimicrobial activity of ovoTF against *Salmonella enterica* depended on the salt composition of the medium, which either favored or interfered with binding of ovoTF to the bacterial surface [50].

The observed anti-chlamydial effects were rather minimal. Nevertheless, they justify further research, especially on the activity of porcine lactoferrin on *C. suis* when inoculated into porcine lactoferrin receptor-expressing swine cells and/or the use of the less expensive ovoTF as an additive to semen extenders. Optimization of the ovotransferrin concentration and the addition of ovoTF activity enhancers could also be examined. Furthermore, although ovoTF seemed to have no effect on the semen cell morphology of spiked semen samples, evaluation of semen quality in the presence of ovoTF needs to be examined thoroughly.

## 4. Materials and Methods

### 4.1. Chlamydia suis, Cell Culture and Transferrins

The effect of transferrins on *C. suis* S45 [51] was examined using McCoy cells (mouse fibroblast cells, CRL-1696 American Type Culture Collection), cultured in Eagle’s minimal essential medium (Thermo Fisher Scientific, Paisley, UK), following standard procedures [52]. McCoy cells were used, as S45 grows significantly better in these cells compared to, for instance, SK6 and Vero cells [53]. Titration of the S45 stock was performed by the method used by Spearman and Karber [54], which determined the TCID_50_.

Ovotransferrin and bLF purified from chicken egg whites and bovine colostrum were purchased from Bioseutica (Fordras, Lugano, Switzerland) and Sigma (Bornem, Belgium), respectively. For each experiment, a fresh stock solution of transferrins (50 mg transferrin per mL cell culture medium) was prepared and filter sterilized (0.22 µm; Merck Millipore, Overijse, Belgium).

### 4.2. Transferrin Cytotoxicity Assay

The putative cytotoxicity of transferrins for McCoy cells was determined by the MTT assay, as described in [38]. The MTT assay is a colorimetric assay for assessing cell metabolic (mitochondrial) activity. Monolayers in 96-well plates (Greiner BioOne, Wemmel, Belgium) were exposed (in duplicate) to zero, 0.5, 1, 2.5, 5 and 10 mg/mL of transferrins in culture medium. At 24 and 48 h, cytotoxicity was determined. The experiment was repeated once.

### 4.3. Effect of Transferrins on C. suis Infectivity and Replication in McCoy Cells

Pre-incubation of *C. suis* with transferrins prior to inoculation allowed us to examine its effect on the infectivity of extracellular bacteria. Bacteria (10^8^ TCID_50_/mL) were incubated at 37 °C for 1 h in McCoy culture medium supplemented with 0.5 mg/mL of ovoTF, 0.5 mg/mL of bLF or 5.0 mg/mL of bLF. Concentrations were selected based on transferrin cytotoxicity results for McCoy cells and earlier in vitro experiments with *C. psittaci* [38]. Next, treated and non-treated (controls) bacteria (100 µL) were inoculated on McCoy cells grown on sterile glass coverslips (*Chlamydia* Trac bottles; Sterilin Ltd., Stone, UK); the monolayers were washed and culture medium was added.

To assess the effect of transferrins on intracellular chlamydiae, McCoy cells grown on sterile glass coverslips were inoculated (100 µL) with *C. suis* S45 (10^8^ TCID_50_/mL), following standard procedures [52]. Monolayers were washed to remove unattached chlamydiae and culture medium with no transferrins (controls), 0.5 mg/mL of ovoTF, 0.5 mg/mL of bLF or 5 mg/mL of bLF was added.

For each of the above-mentioned assays, monolayers were stained at 40 h p.i. and bacterial replication was quantified by Imagen^TM^ *Chlamydia* immunofluorescence staining (Oxoid, Thermo Fisher Scientific, Drongen, Belgium), staining the cell nuclei with DAPI (Life Technologies, Belgium). Quadruplicates were always used. All inoculation trials were repeated once.

### 4.4. Effect of Ovotransferrin on C. suis Spiked Pig Semen

OvoTF was tested as an anti-chlamydial semen additive. First, 3 boar semen samples (artificial insemination center, Flanders, Belgium) were examined with a *C. suis* real-time PCR [55], and blood samples were analyzed using a *C. suis* recombinant MOMP-based ELISA [56] to select one negative boar for spiking assays. Then, 6 mL of fresh, undiluted semen from the selected *C. suis* negative boar was divided into two equal parts. Subsequently, each sample was spiked with 10^8^ TCID_50_ *C. suis* bacteria. Sample one (3 mL) was incubated for 1 h at 37 °C in the presence of 0.5 mg/mL of ovoTF, while sample two (3 mL) was incubated in the same way, adding only cell culture medium (control). Afterwards, 100 µL of the semen samples was inoculated onto McCoy cells [52]. Monolayers were washed to remove unattached bacteria and culture medium, with or without ovoTF (0.5 mg/mL), was added. At 40 h p.i., the replication of *C. suis* was examined using Imagen^TM^ *Chlamydia* immunofluorescence staining. Quadruplicates were used. The experiment was repeated once with semen of another *C. suis*-negative boar.

### 4.5. High Content Microscopy and Image Analysis

A fully automated inverted Nikon Ti widefield fluorescence microscope (Nikon Instruments, Paris, France) was used, equipped with a motorized XYZ stage and filter cube turret and shutters. Samples were magnified with a 40× Plan Fluor oil objective (numerical aperture of 1.3) and images were acquired with an Andor Ixon EM-CCD camera, yielding a pixel size of 0.276 µm/pixel. To obtain a representative sample of each condition with minimal edge effects, three separate, but sufficiently central, regions were chosen per slide. Per region, a 5-dimensional hyperstack was recorded, consisting of 16 fields (acquired as a mosaic of 4 × 4 juxtaposed fields), 5-7 Z-slices (separated by 1 µm) and 2 channels (DAPI and FITC).

To analyze the multidimensional image data sets, a dedicated macro script for FIJI image analysis freeware (http://fiji.sc, version 1.53c [57]) was used as described before [53,58]. Briefly, the analysis consists of a stepwise segmentation of regions of interest (nuclei and *Chlamydia* inclusions), followed by a quantification of their size and number. Before commencing segmentation, hyperstacks were projected along the Z-axis according to the maximum intensity. Then, putative *Chlamydia* inclusions were segmented. To this end, the FITC channel image was convolved with a Laplacian filter to enhance the focal signals (spots), which were automatically thresholded using the Isodata algorithm. Only spots larger than 3 pixels were retained. Subsequently, nuclei were segmented in the DAPI channel after Gaussian smoothing using the Isodata auto threshold algorithm, followed by a binary watershed procedure to separate touching nuclei. Finally, cellular regions of influence were determined by Voronoi tessellation on the binary mask of nuclear regions detected in the image. Per cellular region of influence, the mean spot area (a measure for inclusion size per field), mean spot number (a measure for inclusion number per field) and mean fluorescent area (total inclusion size per field, a proxy for the overall replication) were determined.

### 4.6. Statistical Analysis

The mean and standard error were calculated for the number of inclusions, inclusion size and overall replication. Assumptions of normality and homoscedasticity were checked, and data were transformed in case of non-normality or heteroscedasticity. For all parameters, transferrin concentrations were compared using one-way ANOVA, followed by Tukey HSD post hoc tests (*p* < 0.05). All data were analyzed using TIBCO Spotfire S+^®^ 8.2 (Tibco Software Inc., Palo Alto, CA, USA).

## Data Availability

The script for analysis is available upon request.
